# Exclusive breastfeeding and infantile colic: a cohort study

**DOI:** 10.1186/s13006-026-00816-x

**Published:** 2026-02-12

**Authors:** T. T. Duong Doan, Colin Binns, Yun Zhao, Ngoc Minh Pham, Andy Lee, T. P. Hoa Dinh

**Affiliations:** 1 College of Health Sciences, VinUniversity, VinUniversity, Vinhomes Ocean Park, Gia Lam District, Hanoi, 100000 Vietnam; 2 School of Population Health, Curtin University, Bentley Western, 6102 Australia; 3 Faculty of Social Sciences, Behavior and Health Education, Hanoi University of Public Health, 1A Duc Thang, Bac Tu Liem, Hanoi, 100000 Vietnam

**Keywords:** Exclusive breastfeeding, Colic, Cohort, Infant

## Abstract

**Background:**

Infant colic or excessive crying in early life is a common symptom without a clear understanding of etiology and management. Breastfeeding has both short and long-term health benefits for mothers and children.

**Methods:**

This study was a secondary analysis of a prospective intervention trial on breastfeeding promotion in Vietnam. A total of 856 mother-infant dyads were included to investigate the association between sources of nutrition and infant colic. Data on the exclusive breastfeeding were collected twice in 1-week and 4-weeks after delivery. Colic cases were defined using Rome IV criteria.

**Results:**

The prevalence of colic was 17.2% (95%CI: 14.6 to 19.7%) among infant 3–21 weeks of age, peaked at 7–9 weeks (23.7%, 95%CI: 17.4 to 30.0%), and almost dismissed after 12 weeks (3.6%, 95%CI: 0.08 to 7.2%). In multivariable analyses, compared with mixed feeding, infants under 10 weeks who had exclusive breastfeeding since birth were significantly less likely to have colic (aOR = 0.33; CI: 0.18 to 0.60) after controlling for maternal age, educational level, pre-pregnancy body mass index, parity, mode of delivery, sex assigned at birth of infants, gestational weeks at the delivery, birth weight, group randomization, and infant age. The odds of having colic were also lower among infants who received some formula feed during the first week but then received breastmilk exclusively afterward compared with those received mix feeding since birth.

**Conclusions:**

We observed the lower prevalence of colic among exclusive breastfeeding infants compared to those had mixed feeding. Further studies should be undertaken to examine the relationship between exclusive breastfeeding and infant colic.

## Background

Infant colic or excessive crying in early life is a common symptom that is present in 14% to 30% of infants regardless of ethnicity [1]. The mean cry duration was about two hours and “peaked” in the first 6 weeks and mostly resolved after 12 weeks of age [2, 3]. The Rome IV consensus group defines infant colic as “recurrent and prolonged periods of crying, fussing, or irritability in infants under five months, without obvious cause and could not be resolved or prevented by caregivers, and with no evidence of failure to thrive, fever, or illness”. For clinical research, besides the aforementioned criteria, colic infants had both of the following: (1) cried or fussed for three or more hours a day during three or more days in seven days by caregiver interview and (2) 24-hour behavior prospective diary with the excessive crying infants group to confirm [4].

Infants with colic are described as fussier, present more sleeping problems, and are breastfed less when compared to the non-colic group [5]. Excessive crying could lead to 20% of pediatric consultations [1]. Both mothers and fathers of infants with colic were more likely to have problems with stress, depression, anxiety, and bonding [6]. Studies have found that infants with colic were more likely to have gastrointestinal disorders, adaptive problems in the preschool period, early weaning, attention deficit hyperactivity disorder, migrants, and other behavioral issues in the later stage of life [1, 7, 8]. Other studies suggested that infants with colic have no adverse effects regarding child behaviors, regulatory abilities, temperament, or family functioning in the medium term [9, 10].

The etiology of infant colic remains unresolved and is thought to be multifactorial; however, a growing body of evidence suggests that gut microbiota, inflammation, and, more recently, the microbiota-gut-brain axis contribute to the development of the condition [11–13]. Excessive crying was reported higher in a group of small gestational age or premature births [14, 15], firstborn children [10, 15], low birth weight [15], siblings of colicky children [10] among mothers who have preexisting maternal anxiety, alcohol consumption, or shift work during pregnancy [15, 16]. Cow milk proteins may trigger colic symptoms. In some studies, symptoms of colic were improved in infants who were given formula-free of cow’s milk proteins or breastfed by mothers who avoided cow’s milk [17, 18]. However, other studies found that breastfeeding did not have a protective effect on the development of infant colic [16]. Mothers of infants with infant colic had a low score of breastfeeding self-efficacy and breastfeeding success [19], duration of exclusive breastfeeding was shortened [20]. More long-term prospective studies are required in different cultures.

The health benefits of breastfeeding for infants and mothers in the short-term and long-term are extensive [21, 22]. The World Health Organization (WHO) and United Nations International Children’s Emergency Fund (UNICEF) has developed the Global Strategy for Infant and Young Child Feeding which recommended “Infants should be exclusively breastfed for the first six months of life to achieve optimal growth, development and health” [23] and a number of documents such as guidelines on protecting, promoting, and supporting breastfeeding in health facilities and the Global Breastfeeding Scorecard for framing policy relevance [24]. Many studies of infant colic have been conducted in countries with a long history of formula milk consumption using predominantly Caucasian samples [2, 3, 16, 25]. We, therefore, used a cohort with prospectively collected data to investigate the association between exclusive breastfeeding and infant colic in Vietnam, a Southeast Asia country without a history of cow milk consumption. Our study was based on the hypothesis that the prevalence of colic among exclusive breastfed infants would be lower than among those who were mixed or formula-fed. The hypothesis was derived from the nutritional and immunological superiority of breastmilk compared to formula, including enhancing mother-child bonding from breastfeeding.

## Methods

### Design and setting

This study utilized data obtained as part a randomized controlled trial which used a mobile application to improve exclusive breastfeeding among mothers who had cesarean sections. The study recruited and followed mothers who had singleton pregnancy from 24 to 36 weeks of gestation when they visited the two public hospitals in Hanoi, Vietnam, for antenatal care started from 09/03/2020. Mothers who had high-risk pregnancies were excluded. Eligible mothers were randomized into the control or intervention group to test the effectiveness of the mobile application on breastfeeding outcomes. The intervention group received notifications on breastfeeding, while the control group was provided information on maternal and child health care in general. Both could access the library in the mobile application, which had information on breastfeeding and maternal care. The following interviews were conducted for 1-week, 4-week, 4-month, and 6-month after delivery by telephone and completed in July 2021. The first call was based on the estimated due date provided by mothers at the enrolment. Mothers were contacted five times on five consecutive days of each round and re-contacted in the next round if they had not yet completed the questionnaire. The age of infant at 4-week interview, therefore, ranged from 3 to 24 weeks. Details of the study design have been described and published previously [26].

A total of 1260 mothers were enrolled during pregnancy, 1044 finished both the 1-week and 4-week phone interviews (82.9%). Numbers of individuals and reasons for non-participation at each stage of study have been reported [27]. We excluded 166 (15.8%) mothers who reported their infants had health issues because colic was defined as “without obvious cause and could not be resolved or prevented by caregivers, and with no evidence of failure to thrive, fever, or illness”, 13 infants over 5 months of age (> 21 weeks)(Rome IV criteria for the diagnosis of infant colic) [4]. Besides breastmilk and formula, a very small percentage of infants were fed with other food such as porridge (0.2% at 1 week) or fruit juice (0.8%) at 4-week interviews, those cases were also excluded from the analysis. The final dataset included in the analysis was 856 infant-mother dyads.

### Dependent variable

The dependent variable was infant colic. In the 4-week postpartum interview, mothers were asked questions on the average total amount of crying in hours a day in the previous week and the number of days he/she cried or fussed more than three hours in the previous week. Excessive crying was defined as crying for three or more hours a day during three or more days in the past week, following the criteria of the Rome IV consensus group [4].

### Independent variables

Exclusive breastfeeding was the variable of interest and was based on self-reports by mothers twice: a week and 4 weeks postpartum interview. Mothers were asked, “How did you feed your baby since birth/the last interview?” and “What else did you feed your baby since birth/the previous interview?”. If a mother fed her newborn with breastmilk only (either by breastfeeding or by spoon/bottle) and nothing else except vitamins and oral rehydration salts at both times, she was classified as exclusive breastfeeding since birth according to the definition of the World Health Organization [28]. As most mothers fed their infant with formula milk while waiting for their milk to come (in the first week) and then with breastmilk only or mixed with formula (in the 4-week), exclusive breastfeeding at the 4-week interview was also of interest. For all analyses, the independent variable was the source of infant nutrition (exclusive breastfeeding vs. mixed feeding) at the 4-week postpartum interview (a duration from 1-week interview until 4 week-interview) and since birth, while the outcome, colic, was defined at the 4-week postpartum interview.

Other covariates known to affect exclusive breastfeeding or/and infant colic were included in the analysis including group (intervention: receiving information on breastfeeding vs. control: maternal and child health care in general only through a mobile application), maternal age (< = 35 vs. >35 years old), maternal educational level (high school or lower vs. undergraduate or upper), pre-pregnancy body mass index (BMI, underweight < 18.5, normal weight 18.5–23.0, overweight or obese > 23.0), parity (first vs. second or more child), mode of delivery (cesarean section or vaginal delivery), sex assigned at birth of the infant (girl or boy), gestational weeks at the delivery (preterm less than 37 weeks), birth weight (low birth weight < 2500, normal birth weight 2500 -<4000, macrosomia > = 4000), and infant age in weeks [10, 14–16, 29, 30]. Maternal age, educational level, and pre-pregnancy BMI were collected during pregnancy. Parity, delivery mode, and sex assigned at birth of the infant were collected a week after delivery. Other variables were collected a month after delivery. Delivery mode, sex assigned at birth of the infant, birth weight, and birthdate were checked with hospital records. Discordance values were rechecked with mothers by telephone, and missing values were replaced from the hospital records in cases of unable to contact mothers. The age of the infant was extracted from the birthdate reported by mothers and the interview date. The ages were regroup into 3–6 weeks, 7–9, 10–12, and 13–21 weeks which followed work done by Wolke, Dieter, et al. (2017) for colic infants [2].

### Statistical analysis

Statistical analyses were performed using the Statistical Package for Social Science (SPSS, IBM Corp. Released 2017. IBM SPSS Statistics for Windows, Version 25.0. Armonk, NY: IBM Corp). Continuous and categorical variables were reported as mean (standard deviation, SD) and frequency (%), respectively. The overall prevalence of colic was calculated; similar calculations provided the prevalence of colic among exclusively breastfed and mixed-fed infants by age groups. We assessed the association between infantile colic and breastfeeding by fitting infantile colic using a logistic regression model. We estimated odds ratios (OR) with 95% confidence intervals (CI) for infant colic. We adjusted all analyses for the covariates known to affect exclusive breastfeeding and infantile colic in the model.

## Results

The characteristics of 856 mothers and infants included in the analysis were presented in Table 1. The mean age of mothers was 27.8 years (SD 4.6), and 7.7% were more than 35 years old. About 56.7% of mothers obtained an undergraduate or higher degree of education, 21.4% were underweight, and 9.8% were overweight or obese before pregnancy. Of the births, 46.2% were the first child, 4.1% were preterm, 50.8% were born by cesarean section, and 55.8% were boys. The mean age of the infants at the 4-week interview to collect information on colic was 7.4 (SD 4.2, min 3 weeks), 56.6% at 3–6 weeks, 20.4% at 7–9 weeks, 10.1% at 10–12 weeks, and 12.8% at 13–21 weeks (See Table 1).

**Table 1:** Characteristics of mothers and infants (*N* = 856)

Characteristics	Mean (SD)
	Or *n* (%)
Maternal age: year, mean (SD)	27.8 (4.6)
<=35 years old	790 (92.3)
> 35 years old, n (%)	66 (7.7)
Maternal education	
Highschool or lower, n (%)	371 (43.3)
Undergraduate or upper, n (%)	485 (56.7)
Maternal BMI before pregnancy: kg/m^2^, mean (SD)	20.2 (2.3)
Underweight (< 18.5), n (%)	183 (21.4)
Normal weight (18.5–23.0), n (%)	589 (68.8)
Overweight or obese (> 23.0), n (%)	84 (9.8)
Parity	
First child, n (%)	395 (46.2)
Second child or higher, n (%)	461 (53.8)
Gestational age at birth: week, mean (SD)	38.8 (1.2)
Preterm birth (< 37 weeks), n (%)	35 (4.1)
>= 37 weeks, n (%)	821 (95.9)
Cesarean section delivery, n (%)	
No, n (%)	422 (49.2)
Yes, n (%)	434 (50.8)
Birthweight: gram, mean (SD)	3,219 (409)
Low birth weight (< 2500), n (%)	41 (4.8)
Normal birth weight (2500 -<4000)	785 (91.7)
Macrosomia ( > = 4000), n (%)	30 (3.5)
Sex of the infant	
Girl, n (%)	380 (44.4)
Boy, n (%)	476 (55.6)
Age of the infant: week, mean (SD)	7.4 (4.2)
3–6 weeks, n (%)	483 (56.4)
7–9 weeks, n (%)	177 (20.7)
10–12 weeks, n (%)	86 (10.0)
13–21 weeks, n (%)	110 (12.9)
Source of nutrition at 1-week interview (since birth till 1st week)	
Exclusive breastmilk	207 (24.2)
Mixed feeding	618 (72.2)
Formula	31 (3.6)
Source of nutrition at 4-week interview (since the last interview)	
Exclusive breastmilk	417 (48.7)
Mixed feeding	429 (50.1)
Formula	10 (1.1)
Source of nutrition from birth until 4-week interview	
Exclusive breastmilk	172 (20.1)
Mixed feeding	681 (79.6)
Formula	3 (0.4)
Group	
Control, n (%)	439 (51.3)
Intervention, n (%)	417 (48.7)

Abbreviations. BMI: body mass index; SD: standard deviation

Mixed feeding peaked at 72.2% in week one postpartum and declined to 50.1% in the 4-week interview. Infants who were only fed formula milk accounted for a small percentage: 3.6% at 1-week, 1.1% at 4-week interviews, and overall, 0.4% since birth. The exclusive breastfeeding prevalence was 24.2% at 1-week and increased to 48.7% at 4-week interview, and 20.1% since birth.

**Fig. 1:**
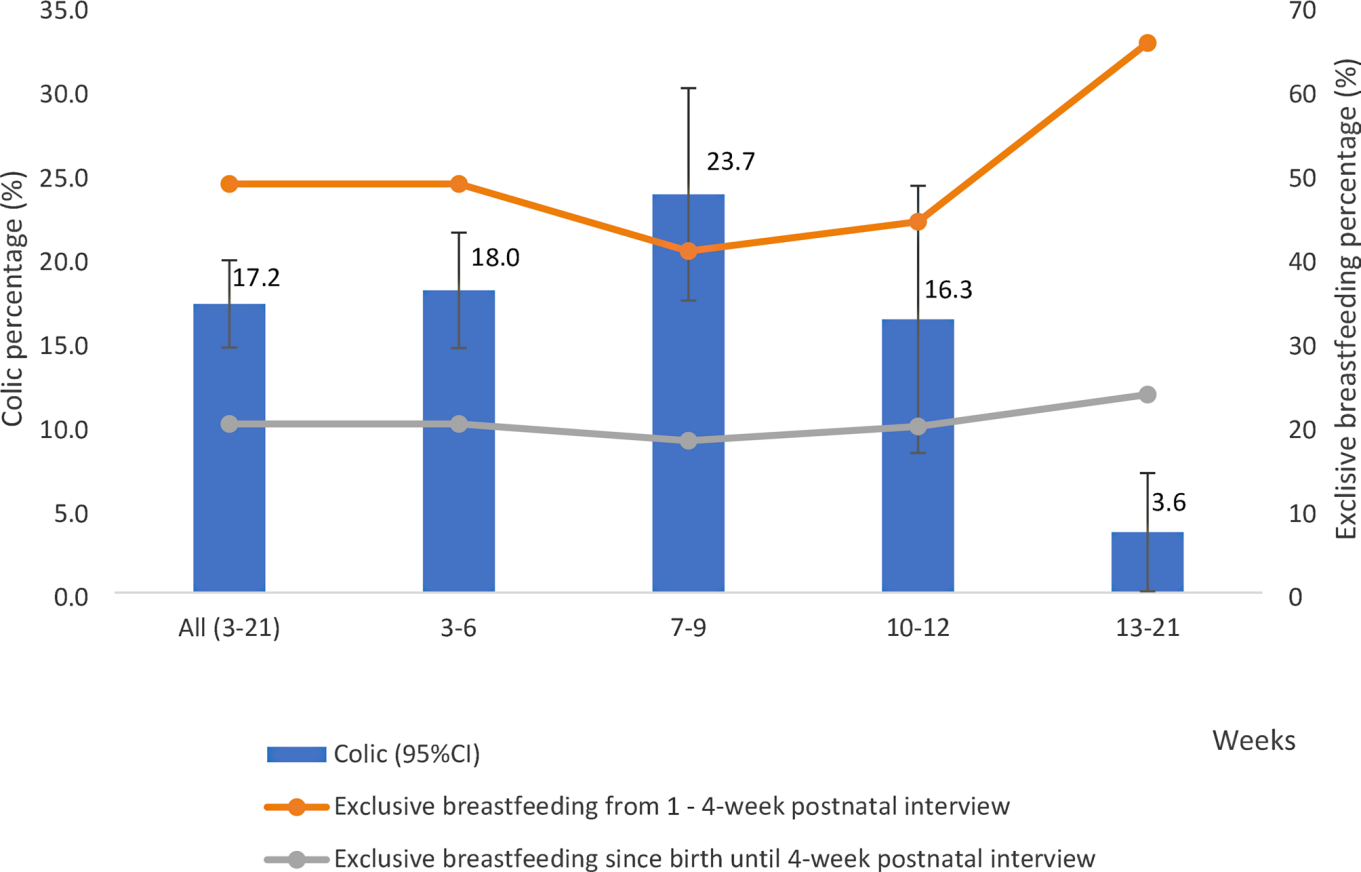
Infant colic by age of the child and exclusive breastfeeding (*N* = 856)

The prevalence of infantile colic among infants 3–21 weeks of age was 17.2% (95%CI: 14.6 to 19.7%). The symptoms peaked around 7–9 weeks (23.7%, 95%CI: 17.4 to 30.0%) and were mostly dismissed by the age of 12 weeks (3.6%, 95%CI: 0.08 to 7.2%), which was in contrast with the exclusive breastfeeding prevalences both at the 4-week interview and since birth: the lowest was at 7–9 weeks, and the highest was at 12–21 weeks (Fig. 1).

Table 2 presents the infant colic prevalence by age group and source of nutrition. The highest prevalences were found among those 7–9 weeks of age and mixed feed (30.7%). No infant who had been exclusively breastfed since birth had colic symptoms after 9 weeks of age.

**Table 2:** Infant colic prevalence by age and being breastfed exclusively (*N* = 856)

Age of infant (week)	Source of nutrition at 4-week interview		Source of nutrition since birth	
	Breastmilk only, *n* (%)	Mixed feeding, *n* (%)	Breastmilk only, *n* (%)	Mixed feeding, *n* (%)
3–6	15 (6.3)	70 (28.7)	10 (10.1)	75 (19.4)
7–9	9 (12.3)	31 (30.7)	4 (12.1)	36 (25.4)
10–12	3 (7.7)	10 (21.7)	0 (0.0)	13 (19.1)
13–21	1 (1.4)	3 (8.3)	0 (0.0)	4 (4.8)
All	28 (6.7)	114 (26.7)	14 (8.0)	128 (18.8)

Note: Subgroup sizes are small by age groups, particularly among infants after 10 weeks of age

Because of small subgroup size of infants 10 weeks and upper, a logistic regression model was performed for those 3–9 weeks only. Infants under 10 weeks who had exclusive breastfeeding since birth had a lower odds to exhibit colic, relative to those who had mixed feeding (crude OR = 0.37, 95%CI: 0.21 to 0.65 in bivariate analysis and adjusted OR = 0.33, 95%CI: 0.18 to 0.60) in a multivariable logistic regression model adjusted for maternal age, maternal education, pre-pregnancy body mass index, parity, gestational age at birth, mode of delivery, birthweight, sex assigned at birth of infant, age of the infant, and random group, Table 3). The odd of having colic among exclusively breastfed infants at 4-week interviews were lower compared to those with mixed feeding (crude OR = 0.19, 95%CI: 0.12 to 0.30 for the bivariate analysis and adjusted OR = 0.19, 95%CI: 0.12 to 0.30 for the multivariable logistic regression model). Besides the source of nutrition, none of the co-variables were statistically significant either in a model with exclusive breastfeeding since birth or at 4-week interview, except age of infants as previously mentioned.

**Table 3:** Association between infant colic and source of nutrition among infants 3–9 weeks

Outcome	Infant colic		cOR (95% CI)	p*	aOR (95% CI)	p**
	No, *n* (%)	Yes, *n* (%)				
Source of nutrition since birth (*n* = 659)				**0.006**		**0.003**
Mixed feeding	415 (78.3)	115 (21.7)	1		1	
Breastmilk only	115 (89.1)	14 (10.9)	**0.44 (0.24 to 0.79)**		**0.40 (0.22 to 0.74)**	
Source of nutrition at 4-week interview (*n* = 654)						
Mixed feeding	242 (69.7)	105 (30.3)	1	**< 0.001**	1	**< 0.001**
Breastmilk only	283 (92.2)	24 (7.8)	**0.20 (0.12 to 0.31)**		**0.19 (0.11 to 0.30)**	

Abbreviations. CI: confidence interval; OR: odds ratio, *Based on bivariate logistic regression model, **Based on multivariable logistic regression model adjusted for maternal age, maternal education, pre-pregnancy body mass index, parity, gestational age at birth, mode of delivery, birthweight, sex of infants, age of the child, and randomized group, aOR=Adjusted OR, cOR=Crude OR

## Discussion

Our study examined the association between the source of nutrition and infant colic in Vietnam. Results showed consistently lower prevalences of colic among exclusively breastfed infants compared with those who received mixed feeding at all age groups. Notably, exclusive breastfeeding was associated with a lower odd of having colic among infants under 10 weeks by 67% to 81% on average, relative to mixed feeding. Our data agree with several previous cohort studies conducted in Ireland [31], the United Kingdom (the Isle of Wight) [32], the Netherlands [33], Sweden [34] and Thailand [35] where the lower odds of being colic among exclusive breastfeeding infants over mixed feeding were documented. However, some other national representative studies in the United Kingdom (Sheffield), Canada, or Norway reported no association between infant colic and exclusive breastfeeding [5, 16, 30]. The discrepancy may be in part due to lack of clear definitions of exclusive breastfeeding and infant colic or the use of broad questions to assess colic. For example, only one study in Canada had a clear definition of exclusive breastfeeding and used criteria to assess infant colic [16]. Other studies only asked a mother a single question: if their child had colic or not [30, 36].

Our findings suggested that the odds of having colic were lower among infants who received some formula feed during the first week but then received breastmilk exclusively afterward compared to those who received mixed feeding. This could be explained by the peak prevalence of colic among infants aged 7–9 weeks. Most infants with cow’s milk protein allergy or intolerance develop symptoms before four weeks of age, often within one week after the introduction of a cow’s milk-based formula [37]. Further studies are needed to confirm our results; however, it is an encouragement for mothers to keep exclusive breastfeeding following the WHO recommendations as most of them have some difficulties feeding their infants exclusively during the first week. Changing the diet of the infants to exclusive breastfeeding to treat colic also should be considered and test its effectiveness in further clinical trials.

The strong effect sizes could be explained by mothers changing their feeding methods, from exclusive breastfeeding to mix-feeding to response to the colic. A previous systematic review indicated that mothers who had excessive crying infants had insufficient or harmful breastmilk, and had lower confidence in breastfeeding, and being pressured from others to stop breastfeeding [38]. Further studies should be designed to understand the impact of changing feeding methods to colic. While the etiology of infant colic remains inconclusive, the unusually strong effect sizes, however, could be possible residual confounding or bias. Known covariables contributing to the development of the condition such as gut microbiota, inflammation, microbiota-gut-brain axis, maternal anxiety, maternal diet including cow milk, and shift work during pregnancy are needed to be included in further studies [11–13, 15, 16].

Most of the studies on infant colic and the source of nutrition conducted from milk-consumption countries and mostly from White people, of whom organic causes (e.g., protein intolerance, lactose malabsorption) were responsible for only a small subgroup of cases of colic [16, 25]. Cow’s milk protein allergy/cow’s milk protein intolerance in infancy were about only 2–5% [37]. Vietnamese population may have a higher incidence of lactose malabsorption than the traditional “non-milking” attitude [39]. A bigger contribution of cow’s milk intolerance should be expected [17]. A study in Thailand, a Southeast Asian country that shares a border with Vietnam, also found that the odds of having colic among exclusively breastfeeding infants for the first eight weeks of life was lowers compared to those with mixed or formular feedings [35].

The are several limitations that need to be considered when interpreting the results of this study. The colic prevalence could be underestimated because of loss of follow-up and the exclusion of infants who had health problems since birth. Mothers who had an infant with colic may be more likely to experience mental health issues and refuse to be interviewed [6]. Following the definition of colic, we exclude infants who had health problems; however, it was defined since birth since colic was assessed within seven days, which could eliminate colic cases without the presence of failure to thrive, fever, or illness. We lacked a 24-hour behavior diary to confirm that infants cried or fussed for 3 or more hours/day during 3 or more days in a week as reported by mothers. Consequently, the recall bias due to parents being stressed or anxious led to overestimating the length of crying and higher prevalence of infant colic. Feeding practices may have changed in response to infant crying which we were not measured. Admittedly, because the participants in our study were, on average, well-educated and relatively affluent, findings may be generalized to similar urban, relatively educated populations and in a setting with low formula-only feeding prevalence. Nevertheless, the internal validity of our study is high because of the inclusive nature of the study population and high response rates. We also collected information on types of feeding twice, a week and 4-weeks postpartum interview and having cross-checked questions if an infant having feed with another food/fluid to ensure an infant was breastfed exclusively. Our findings are exploration and hypothesis-generating due to and observational secondary analysis design, strong effect sizes, and potential reverse causality. Further studies should be undertaken on a larger scale and using behavior diaries to confirm the effect of exclusive breastfeeding or the source of nutrition on infant colic.

## Conclusion

In this study 17.2% of infants aged under four months had symptoms of colic. The study added to the evidence that exclusive breastfeeding may confer protection against infant colic, especially in communities with high cow’s milk intolerance. Our findings are exploration and hypothesis-generating. Further studies should be undertaken on a larger scale and using behavior diaries to confirm the effect of exclusive breastfeeding or the source of nutrition on infant colic.

## Data Availability

The database used for this study is part of a trial in Hanoi, Vietnam. The data can be assessed by contacting Duong Doan ( [doan.ttd@vinuni.edu.vn](mail to: doan.ttd@vinuni.edu.vn)), the corresponding author.

## Abbreviations

BMI: Body Mass Index
CI: Confident interval
OR: Odds ratio
SD: Standard deviation
